# Nursing home care educational intervention for family caregivers of older adults post stroke (SHARE): study protocol for a randomised trial

**DOI:** 10.1186/s13063-018-2454-5

**Published:** 2018-02-09

**Authors:** Carolina Baltar Day, Carla Cristiane Becker Kottwitz Bierhals, Naiana Oliveira dos Santos, Duane Mocellin, Mariane Lurdes Predebon, Fernanda Laís Fengler Dal Pizzol, Lisiane Manganelli Girardi Paskulin

**Affiliations:** 1 Nursing School, Nursing Graduate Program, Universidade Federal do Rio Grande do Sul (UFRGS), São Manoel Street, 963, Rio Branco, Porto Alegre, 90620110 Rio Grande do Sul Brazil; 2 Nursing Department, Franciscan University Center (UNIFRA), Santa Maria, Brazil; 3 Nursing Department, Hospital de Clínicas de Porto Alegre (HCPA), Porto Alegre, Brazil

**Keywords:** Randomized controlled trial, Nursing, Stroke, Aged, Caregivers, Home care services

## Abstract

**Background:**

Family caregivers of aged stroke survivors face challenging difficulties such as the lack of support and the knowledge and skills to practice home care. These aspects negatively influence the caregivers’ burden and quality of life, the use of health services, and hospital readmissions of the stroke survivor. The aim of this research is to describe an educational intervention focused on family caregivers of stroke survivors for the development of home care in the south of Brazil.

**Methods:**

A randomized clinical trial with 48 family caregivers of stroke survivors will be recruited and divided into two groups: 24 in the intervention group and 24 in the control group. The intervention will consist of the systematic follow-up by nurses who will perform three home visits over a period of 1 month. The control group will not receive the visits and will have the usual care guidelines of the health services. Primary outcomes: burden and quality of life of the caregiver. Secondary outcomes: functional capacity and readmissions of the stroke survivors; the use of health services of the stroke survivors and their family caregivers. Outcomes will be measured 2 months after discharge. The project was approved in April 2016.

**Discussion:**

This research offers information for conducting educational intervention with family caregivers of stroke survivors, presenting knowledge so that nurses can structure and plan the actions aimed at the education of the family caregiver. It is expected that the educational intervention will contribute to reducing caregiver burden and improving their quality of life, as well as avoiding readmissions and inadequate use of health services by stroke survivors.

**Trial registration:**

ClinicalTrials.gov, ID: NCT02807012. Registered on 3 June 2016. Name: Nursing Home Care Intervention Post Stroke (SHARE).

**Electronic supplementary material:**

The online version of this article (10.1186/s13063-018-2454-5) contains supplementary material, which is available to authorized users.

## Background

Stroke is the third leading cause of death in developed countries and the second in developing countries such as Brazil [1]. In the southern region of the country, this disease presents the highest rates of hospital admissions [2]. In older adults, stroke is the second cause of death and the most prevalent cerebrovascular disease [1].

In Brazil, approximately 50% of older adults affected by stroke survive. These adults require assistance for daily activities and they are cared for by their own family at home [3]. The family caregivers face difficulties related to the instrumental, emotional and financial support and also the lack of knowledge and skills to perform the activities of care [4]. Support programs for caregivers after hospital discharge have demonstrated beneficial effects when structured in training interventions for family caregivers so that stroke survivors can receive proper care at home [5, 6]. These interventions are associated with the reduction of the caregiver’s burden and the improvement of their quality of life (QofL) [7, 8].

A structured model of home care for caregivers of stroke survivors after hospital discharge is non-existent in Brazil. However, because of the aging population and the need to reduce hospital costs, it is necessary to discuss national health policies directed toward home care [9].

Currently, discharge planning with adequate transition of care is not a reality in most hospital institutions and the patients return home without receiving adequate orientation and information on post-discharge care. These aspects may leads to major risks for readmission in hospitals and demands on the emergency services [9–12].

In Brazil, the home care policy determines that the health professionals, who are part of the home care teams, provide assistance for the users, being responsible for training the caregivers and involving them in the care [13, 14]. However, although Brazilian public health policies define the formation of a health care network, they present fragility, lack of transition of care and inefficient health services in home care for stroke survivors and their caregivers [15]. As a consequence, the responsibility for the care of stroke survivors rests with the family and with an informal network of community members.[16].

Care and assistance for daily life activities of the stroke survivors is often unexpected. Family members in this situation suddenly find themselves without previous training [15, 17, 18]. This context contributes to the high level of caregiver burden [3], also influencing the worsening of their QofL [19]. Furthermore, without guidance, care for the aged cannot be expected to be understood and performed correctly by the caregiver [20], contributing to the consequent hospital readmissions of the older adults and the complications of negligent home care [21].

The acquisition of knowledge by the caregiver is not arbitrary, but rather acquired through the guidance of a health professional at the time of hospital discharge or when returning to the community [3]. Some studies have described models of educational intervention directed to caregivers of stroke survivors at home after discharge, using telephone or educational groups conducted by nurses or multidisciplinary teams [8, 22–24]. They focus on the training of technical skills for care, resulting in beneficial results for the older adults and their caregivers [5, 25].

An integrative review of Brazilian studies on family caregivers of the aged proposes interventions of social, emotional and psychological support as a form of assistance and improvement of the QofL of the caregivers at home [26]. In home care, the nurse is the professional who has the skills to educate the family caregiver of the stroke survivor in the face of this new reality [27]. In addition, it plays a fundamental role in the orientation and education of these patients and their families, both at the time of hospital stay and when discharged to return home.

This research offers information for conducting educational intervention with family caregivers of stroke survivors, presenting knowledge that nurses can structure and plan the actions aimed to the education of the family caregiver, as well as to assist them in their care activities. In addition, it shows that the adequate preparation of these caregivers positively influences their burden and QofL, as well as avoiding hospital readmissions or inadequate use of health services.

The protocol offers support for the strengthening and effective implementation of home care services (HCS) as a modality of care. Moreover, it can be used by hospital transition teams that continue home care after discharge, in an attempt to avoid rehospitalization; by primary care services, in which physicians and nurses make home visits (HVs) to the families; and in the support of HCS already structured or structuring.

## Methods

### Aim

To describe an educational intervention focused on family caregivers of the stroke survivors for the development of care at home (SHARE) in the south of Brazil.

### Objectives of the research

1. To determine the effect of the SHARE intervention on the burden of care and QofL of family caregivers of stroke survivors compared to conventional care
2. To evaluate the stroke survivors’ hospital readmissions and the use of the health services by them and their caregivers after the SHARE intervention

### Hypotheses

1. The family caregivers who will receive the intervention will have a better QofL and less burden when compared to the caregivers allocated in the control group (CG)
2. The stroke survivor in the intervention group (IG) will present greater functional independence and will have fewer hospital readmissions and use of health services than those in the CG

### Design

**Fig. 1:**
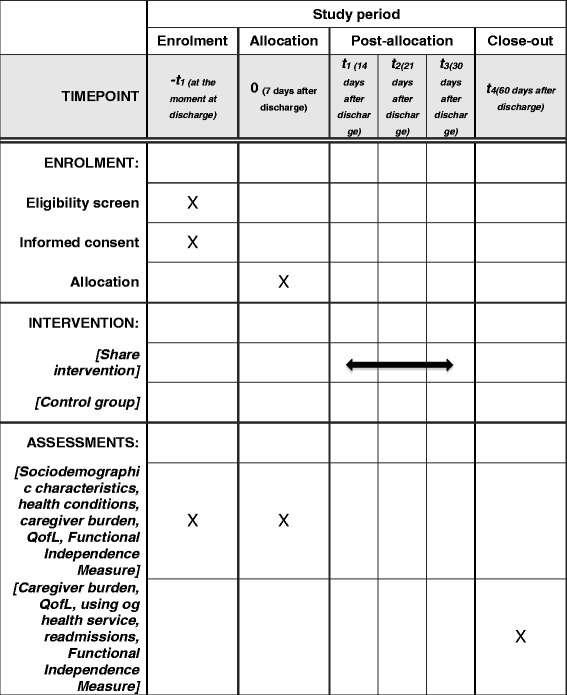
Schedule of enrollment, interventions and assessments of the SHARE intervention

A randomized controlled trial (RCT), blinded and with follow-up (2 months after hospital discharge), designated Nursing Home Care Intervention Post Stroke (SHARE). All stages of the RCT are in accordance with the recommendations of the Consolidate Standards of Reporting Trials (CONSORT) [28]. The protocol study is in accordance with the Standard Protocol Items: Recommendations for Interventional Trials (SPIRIT) Checklist as Additional file 1 and Fig. 1.

### Setting

The locale of recruitment of the participants is the Special Care Unit of Stroke (SCU-Stroke) at a university hospital in the south of Brazil. The SCU is intended to serve patients with a diagnosis of stroke and is part of a clinical hospitalization unit where approximately 10 beds are allocated to stroke patients. In this unit, they are followed up by a multidisciplinary team that provides care information according to the needs of patients and their families. The intervention and follow-up of the participants will be carried out in the homes of the stroke survivors.

### Participants

The study participants will be the family caregivers selected at the time of hospital discharge at the SCU-Stroke. Family caregivers will be considered the persons responsible for the care of the stroke survivor at home. They will reorganize the home for the stroke survivor and perform most of the care. They may or may not have consanguineous ties with the stroke survivor.

### Inclusion criteria

Participants will be included if they meet the following criteria: (1) to be a family caregiver of the aged (60 years old or more) stroke survivor with the first functional sequel from the SCU-Stroke; (2) stroke survivor with a minimum score of 2 on the Modified Rankin Scale (mRankin) at the time of discharge; (3) family caregiver 18 years or older; (4) a family caregiver who provides unpaid care and (5) stroke survivor who resides in the city of the study or another city within a distance of 20 km from the hospital.

### Exclusion criteria

Participants will be excluded if: (1) the stroke survivor resides in a long-term institution; (2) the stroke survivor is included in an HCS; (3) the stroke survivor died during the study and (4) the family caregiver is not interested in participating in the SHARE intervention.

### Recruitment and randomization

The stroke survivors and their family caregivers will be selected during the hospital stay at SCU-Stroke. Those who meet the inclusion criteria and are eligible will be invited to participate in SHARE. Ethical procedures will be guaranteed and participants will be able to discontinue participation at any time in the research. Participants selected will receive a HV for baseline assessment within 7 days after discharge. Participants will be picked up, selected and evaluated by research assistants, who in order to minimize bias, will be responsible for: (1) obtaining the consent of family caregivers and the stroke survivors and (2) sending the participants baseline data for randomization.

The allocation of the participants in the IG or in the CG will be through simple randomization, through a numerical list generated by the website randomization.com. This list has a numerical order, in which each number is already assigned to one of the groups randomly. Thereafter, patients are given a number and their designation as they are included in the study. Randomization will occur after the baseline evaluation of stroke survivors and family caregivers, and will be performed by a trained research assistant who is not involved in the evaluation of the participants nor involved in the SHARE intervention.

**Fig. 2:**
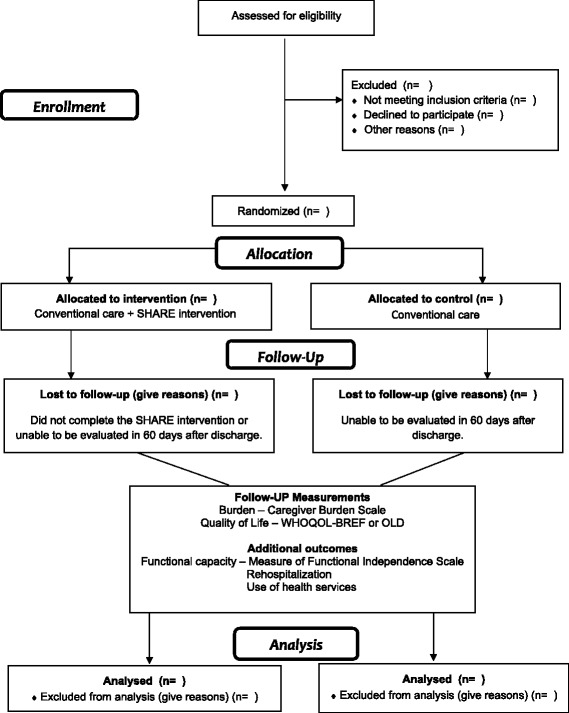
Logistic of the study

The CG will receive conventional care, and the IG will receive the SHARE intervention. The assistant responsible for randomization will inform the interventional nurses which patients are assigned to the IG. The final evaluation of all participants will be performed by the same research assistants in the initial assessment 60 days after the date of the patient’s discharge from the hospital. The logistics of the study are presented in Fig. 2. Regarding the blindness, the trial will have outcomes obtained from blinded evaluators. Study participants will not be blind to the intervention.

### Study treatment

#### Conventional care

The usual care consists of: (1) specialized care for stroke in the SCU-Stroke, carried out by a multiprofessional team (physicians, nurses, pharmacists, nutritionists, physiotherapists, speech therapists, social workers and psychologists), during hospitalization time and at the time of hospital discharge. This team will provide information about stroke survivors’ care and, in addition, will supply educational manuals containing various guidelines on illness, food/nutrition, medication care, mobility, bed placement, among others; (2) follow-up at the hospital outpatient clinic and (3) follow-up in primary health care services.

### Share

In addition to conventional care, SHARE participants will receive intervention that will: (1) guide the family caregiver to assist the stroke survivors in daily living at home and for the use of health services; (2) teach family caregivers about oral or nasoenteric feeding, personal hygiene, bathing, diaper changing, dressing and undressing, transfer, positioning, medication care, handling of devices, such as vesicles and ostomies, if necessary; (3) provide orientation related to the stroke, causes and consequences of the disease, how to prevent a new stroke, what resources the care network offers, and how to access them; (4) provide emotional support to the family caregiver and the stroke survivors and (5) provide educational material from the Health Education collection of Porto Alegre Clinical Hospital (HCPA) [29].

The intervention will be performed by a pair of nurses, trained to offer orientations based on an intervention protocol validated by consensus of specialists [30]. Three HVs will be performed within 1 month after hospital discharge (the first within 14 days, the second by 21 and the third within 30 days). Each intervention visit will last approximately 40 min.

The intervention will be offered verbally using the dialogical/problematizing educational approach developed by Freire in which the educator develops a dialogic process, stimulating the students’ reflective thinking capacity to transform their reality. In this approach an exchange of knowledge can occur in the socio-cultural context of the individual to be educated [31]. The interventionist nurses must: (1) consider the prior knowledge of family caregivers; (2) observe how they develop care according to their socio-cultural environment, experiences and financial resources; (3) encourage family caregivers to think reflectively about how care is being developed; (4) prepare the care intervention with the family caregiver using previous experiences along with the new information offered and (5) respect the knowledge of the family caregiver when performing the care.

### Data collection and outcomes

#### Primary and secondary outcomes

The primary outcomes will be: the caregiver’s burden and QofL, as measured by the Caregiver Burden Scale and by the World Health Organization Quality of Life–BREF (WHOQOL-BREF)_ and the World Health Organization Quality of Life (for caregivers aged over 60 years) (WHOQOL-OLD) [32–34]. Secondary outcomes: functional capacity measured by Functional Independence Measure (FIM) [35]; the hospital readmission of the stroke survivors and the use of health care services by participants after the stroke episode. Primary outcomes and functional capacity will be measured within 1 week after discharge and 2 months after discharge. The outcomes of hospital readmission and use of health services will be evaluated 60 days after discharge (Table 1).

**Table 1:** Outcomes and time of data collection: stroke survivors and their family caregivers

Outcome and instrument	Family caregiver vs. stroke survivors	Data collection time	
		T0 (7 days)	T1 (60 days)
Primary outcomes			
Caregiver Burden Scale	Family caregiver	X	X
World Health Organization Quality of Life (WHOQOL-BREF and -OLD)	Family caregiver	X	X
Secondary outcomes			
Functional Independence Measure (FIM)	stroke survivor	X	X
Hospital readmission (yes/no)	stroke survivor		X
Use of health care services	Family caregiver and stroke survivor		X

### Instruments

#### Caregiver Burden Scale

The Caregiver Burden Scale was adapted and validated to the Brazilian context in 1998, obtaining intra and interobserver reproducibility coefficients of 0.87 and 0.92, respectively [32]. This scale has been used in research to evaluate the caregiver’s burden of caring for stroke patients. It has 22 items divided into five dimensions: general tension, isolation, deception, emotional involvement and environment. It addresses important areas for caregivers such as health, mental well-being, personal relationships, physical burden, social support, finances and the environment [36]. To these questions, answers from 1 to 4 can be attributed, being: 1 – not at all; 2 – rarely; 3 – sometimes and 4 – often. For the measurement of the caregivers burden, the total score is obtained by the arithmetic mean of the values equivalent to the answers of the 22 questions. There is no cut-off point to classify the burden [32].

### WHOQOL-BREF

The instrument World Health Organization Quality of Life-BREF (WHOQOL-BREF) has been used to assess the QofL of the caregivers. This instrument, developed by the World Health Organization (WHO) in a cross-cultural perspective, has been used in national and international research with family caregivers of older adults people with stroke [37–40]. It consists in 26 questions and has two general questions about QofL. It has four domains: “Physical,” “Psychological,” “Social relations” and “Environmental” and there is no cut-off point for the worst or better QofL. The instrument provides a global score and for each of the four domains. The higher the score, the better the perception of QofL [41].

For caregivers aged older than 60 years, in addition to the WHOQOL-BREF, the WHOQOL-OLD module will be applied to evaluate the QofL of the older adults [42]. The OLD has six facets of four items each: “Sensory Abilities,” “Autonomy,” “Past, Present and Future Activities,” “Social Participation,” “Death and Dying” and “Intimacy.” The WHOQOL-BREF and the module OLD were validated for use in Brazil, presenting a Cronbach’s alpha of 0.91 and 0.885, respectively [33, 34].

### Functional Independence Measure

The Functional Independence Measure (FIM) verifies the level of independence of the older adults and was developed in the 1980s by a North American task force organized by the American Academy of Physical Medicine and Rehabilitation and the American Congress of Rehabilitation Medicine [43]. This is an instrument that presents the incorporation of the cognitive evaluation and is being widely used internationally [44].

The scale was translated into Brazilian Portuguese in 2000, obtaining an intraclass coefficient equal to 0.97 [43], and underwent a validation process in 2004 [35]. It is composed of 18 categories, grouped into six dimensions, referring to the motor and cognitive domains: self-care, sphincter control, transfers, locomotion, communication and social cognition. Each task is classified by the score of seven levels (1: total dependency; 2: maximum dependency; 3: moderate dependency; 4: minimal dependency; 5: supervision, stimulation or preparation; 6: independence modified and 7: complete independence) [43]. The total FIM score is obtained by adding the scores of each dimension, the minimum being 18 and the maximum of 126 points [35].

### Other outcomes

Hospital readmissions: yes or no?

Use of services: information will be collected by a questionnaire prepared for the study related to the health care services used by the stroke survivors and their caregivers, as well as the reason for using the services. The services expected to be commonly used by stroke survivors and family caregivers are services from the health public system such as primary health care centers, appointments with nurses and physicians, pharmacies and emergency departments. Furthermore, they may use services from the private health services such as hospitals and appointments with physicians.

### Statistical methods

#### Sample size

The calculation of the sample was estimated by the burden outcome and was based on a study that demonstrated a difference between the caregiver’s burden and an improvement in their QofL after an educational intervention [5]. Considering a confidence level of 0.95, a statistical power of 0.80, a minimum effect size of 0.9 standard deviations between the groups regarding the burden and QofL, and a sample loss of 20%, the sample was calculated in 48 patients. There was a fair division between the two groups, with 24 patients in the IG and 24 patients in the CG. The statistical calculation was performed by the program WinPepi version 11.32 [45].

### Data analysis

The analysis will be performed by intention-to-treat, where data from all participants (stroke survivors and their family caregivers) will be used regardless of the time they participated in the study [46]. Quantitative variables will be expressed as means and standard deviations or median and interquartile range. The Shapiro-Wilk test will be used to assess the distributions of the continuous outcomes. For the qualitative variables, absolute and relative frequencies will be presented. Student’s *t* test, the Mann-Whitney *U* test, Pearson’s chi-square test or Fisher’s exact test according to normality will be used to compare the groups. The linear regression model (quantitative outcomes) and the Poisson regression analysis (qualitative outcomes) will be applied, considering a *p* value of less than 0.20 in the bivariate analysis. A *p* < 0.05 will be considered. The Statistical Package for Social Sciences (SPSS) version 21.0 will be used for analysis.

### Validity and reliability/rigor

All the instruments used in this study were tested for validity and reapplicability in the Brazilian population. In addition, randomization means that there are no systematic biases regarding the subjects’ allocation, since it ensures that it is random, minimizing the influence of confounding variables. Blinding is also critical, as it controls for cointerventions and bias outcome evaluations.

## Discussion

With the aging population, stroke has increasingly affected older adults, causing functional and cognitive consequences that make these people dependent on care for their activities of daily living. Family caregivers will have to perform care activities, often in a sudden way, in addition to dealing with all the changes that this situation generates in the family organization at home. Thus, it is necessary to orientate the family caregivers to perform these activities, so as to offer support that provides greater security and knowledge, and also manages their emotions and reorganizes the family context in front of the dependent stroke survivor.

SHARE is an unpublished study in Brazil, the first one being conducted with family caregivers of older adult stroke survivors. Despite national concerns about the quality of health care, coverage and access to services, the recent proposal for home care and instrumentalization of family caregivers is still incipient. Statistically significant results are expected to demonstrate the reduction of the level of burden and the improvement of the QofL of family caregivers of the older adults stroke survivors. As a secondary outcome, it is also believed that the stroke survivors will improve their functional capacity, reducing the use of the emergency services. Furthermore, the SHARE intervention will promote the use of primary health care services, and hospital readmissions can be avoided.

Several studies have suggested the implementation of interventions that orientate family caregivers in the home context, offering education for the development of technical skills and emotional support, since these are considered more effective in reducing the burden and improving the QofL [47–51]. In addition, they also have a positive effect on reducing the use of services and hospital readmissions, since they improve the abilities of caregivers to perform the activities necessary for the adequate care of the older adults [21, 52].

In this study, outcomes were selected that demonstrate the positive effects of home interventions, both for patients and caregivers, and for the health system. The expected results provide policymakers with information for the strengthening of home care policy. This implies less use of financial resources with hospitalizations and frequent use of emergency health services, both by the stroke survivors and their family caregivers, who may also become sick without adequate support.

It is also considered that the method of this study is robust and adequate to evaluate the effect of educational intervention to family caregivers of the stroke survivors, besides offering important information for the implementation of SHARE in the context of the Brazilian health system. At the moment, the study is in the phase of data collection with an expected end in July 2017.

Possible limitations to this study may include the loss of follow-up due to the death of the stroke survivors, hospitalization in long-term institutions or caregivers exchange, as well as the hiring of formal caregivers. Despite this, the studies developed with this approach in the international context are limited and they are unpublished in the national (Brazilian) context. The present study will contribute to necessary information for future research.

From this RCT it will be possible to provide scientific arguments for the implementation of educational nursing intervention to family caregivers of stroke survivors at home. It is expected that there will be a reduction of the family caregivers’ burden, improvement in their QofL, as well as a decrease in the hospital readmission of the older adults and use of the health services of the stroke survivors and their family caregivers. In addition, this study will provide support for the strengthening of public policies directed to home care and to family caregivers of dependent stroke survivors.

### Trial status

The protocol was registered at ClinicalTrials.gov on 3 June 2016 with identification number NCT02807012. The data recruitment began in May 2016. At the moment, caregivers and stroke survivors have been recruited and the SHARE intervention has been carried out. The recruitment will be completed in December 2017.

## Additional file

Additional file 1: Standard Protocol Items: Recommendations for Interventional Trials  (SPIRIT) Checklist. (PDF 2861 kb)

## Abbreviations

CG: Control group
CONSORT: Consolidated Standards of Reporting Trials
FIM: Functional Independence Measure
FIPE: Fundo de Incentivo a Pesquisa e Eventos
HCPA: Hospital de Clínicas de Porto Alegre
HCS: Home care services
HV: Home visit
IG: Intervention Group
QofL: Quality of life
RCT: Randomized controlled trial
SCU- STROKE: Special Care Unit of Stroke
SHARE: Nursing Home Care Intervention Post Stroke
SPIRIT: Standard Protocol Items: Recommendations for Interventional Trials
SPSS: The Statistical Package for Social Sciences
WHO: World Health Organization
WHOQOL-BREF: World Health Organization of Quality of Life-BREF
WHOQOL-OLD: World Health Organization of Quality of Life-OLD
